# Trophic niche divergence among colour morphs that exhibit alternative mating tactics

**DOI:** 10.1098/rsos.150531

**Published:** 2016-04-13

**Authors:** Matthew S. Lattanzio, Donald B. Miles

**Affiliations:** Department of Biological Sciences, Ohio University, Athens, OH 45701, USA

**Keywords:** colour polymorphism, diet, ecological divergence, resource polymorphism, stable isotope analysis

## Abstract

Discrete colour morphs associated with alternative mating tactics are assumed to be ecologically equivalent. Yet suites of behaviours linked with reproduction can also favour habitat segregation and exploitation of different prey among morphs. By contrast, trophic polymorphisms are usually attributed to morphs exhibiting habitat or prey selectivity. An alternative hypothesis is that habitat variation generates a trophic polymorphism driven by differences in morph reproductive behaviour, the spatial dispersion of morphs in a landscape and their exposure to different prey types. In this scenario, morphs are allowed to vary in habitat or diet selectivity (e.g. specialist or generalist) as they do in behaviour, rather than being assumed to exhibit equivalent levels of ecological specialization. We test this hypothesis using male *Urosaurus ornatus* lizards that exhibit a discrete dewlap colour polymorphism that reflects alternative mating tactics. We found blue morphs specialize on prey at higher trophic levels, yellow males display plasticity in trophic and morphological attributes and orange males are trophic generalists. Our results also demonstrate that morph diet differences are enhanced in resource-limited habitats. We conclude that discrete behavioural morphs may also diverge in morphology and trophic niche. Jointly, these processes may enhance speciation rates in colour polymorphic taxa.

## 1. Introduction

Mechanisms involved in the maintenance of discrete behavioural and morphological polymorphisms within a species include frequency-dependent selection, spatial variation in selection or disruptive selection [1]. Colour polymorphisms in particular are inferred to reflect alternative reproductive tactics and their maintenance requires that each morph has equal fitness in the long term [2]. The integration of divergent hormonal, behavioural and physiological traits to each colour morph by correlational selection may enhance fitness [3], resulting in asymmetries in resource-holding potential and relative dominance of the discrete male morphs [4,5]. Variation in these traits contributes to the relative mating success of each morph [6].

Apart from behavioural differences, morphs are assumed to have similar ecological roles. This assumption is counterintuitive, because behavioural dissimilarities among morphs have been linked to differences in their spatial dispersion within a landscape, the size of their territories or home ranges and the quality of those used habitats [7–11]. For example, in a study on Australian reptiles, Forsman & Aberg [9] show that polymorphic species have individuals capable of exploiting larger areas and occupying a greater diversity of distinct microhabitats than their monomorphic congeners, even after controlling for variation in home range size. It follows that if discrete morphs are capable of exploiting different components of a resource gradient, then their trophic niches should also diverge [12]. This is an underappreciated, yet key ecological consequence of a colour polymorphism.

Trophic polymorphisms have been documented in a diverse array of taxa [13,14]. Although there exists evidence for discrete dietary variation among colour morphs (but see [15–17]), we are aware of only one example that links dietary variation among morphs that exhibit alternative reproductive tactics [18]. Dietary divergence among morphs is expected to arise via one of two mechanisms [17]: morph-specific differences in microhabitat exploitation or morphological constraints (e.g. gape limitation) that influence prey selectivity [16,18,19]. These two hypotheses differ in that the first proposes that a trophic polymorphism is unrelated to diet selection and instead is a function of differences in prey diversity arising from segregation of morphs among different microhabitats. In the first hypothesis, all morphs are predicted to be opportunistic feeders, and thus a trophic polymorphism is simply an indirect outcome of differences in habitat selectivity. By contrast, the second hypothesis assumes the absence of microhabitat segregation among morphs, and thus morphs are predicted to be selective foragers, specializing on different prey types within a shared resource environment.

Alternatively, morph differences in behaviour may coincide with differences in both habitat and prey selectivity among them, but the actual observed degree of trophic differentiation among morphs may depend on resource availability. Under this combination of the first two mechanisms, variation in habitat characteristics (vegetation and structural cover) and prey diversity (number of distinct prey types), morph behaviour and the spatial dispersion of morphs interact to shape a trophic polymorphism. Moreover, this hypothesis also allows morphs to differ in their foraging and microhabitat selection tactics (e.g. specialist or generalist) as they do in behaviour, rather than being assumed to all exhibit the same (or similar) level of habitat or prey specialization. Here, we envision a scenario for a polymorphic lizard in which colour morphs occupy habitats that vary in structural elements such as the relative dominance of woody vegetation (C_3_) versus grasses (C_4_). The distinction between C_3_ and C_4_ vegetation types is based in part on both metabolic differences including the nature of the first stable compound during carbon fixation (i.e. whether it is a three- or four-carbon compound, respectively), as well as clear differences in isotopic signature [20]. Whereas the diet of all morphs may consist of arthropods, the underlying trophic linkages for each morph may involve differences in selectivity for arthropods mainly deriving from woody (i.e. a C_3_-based) versus grassy (C_4_-based) energetic pathways. Although C_3_-based pathways may be preferred over C_4_-based pathways owing to their higher protein and nitrogen content [21], differences in prey selectivity among morphs (e.g. generalist versus specialist) will influence their ability to exploit those preferred prey types. Two predictions emerge from this scenario. In higher-quality habitats dominated by C_3_ vegetation, morphs should exhibit reduced spatial overlap given the abundance of their preferred prey in those habitats. Moreover, all morphs should also be capable of exploiting C_3_-based trophic pathways, regardless of any differences in their prey selectivity or social behaviour. By contrast, C_4_-dominated habitats would favour both greater spatial overlap and trophic dissimilarity among morphs. Because morphs differ in mating and territorial behaviour, we also predict that only aggressive morphs should monopolize preferred C_3_ dietary resources when they are limiting [11].

**Figure 1.**
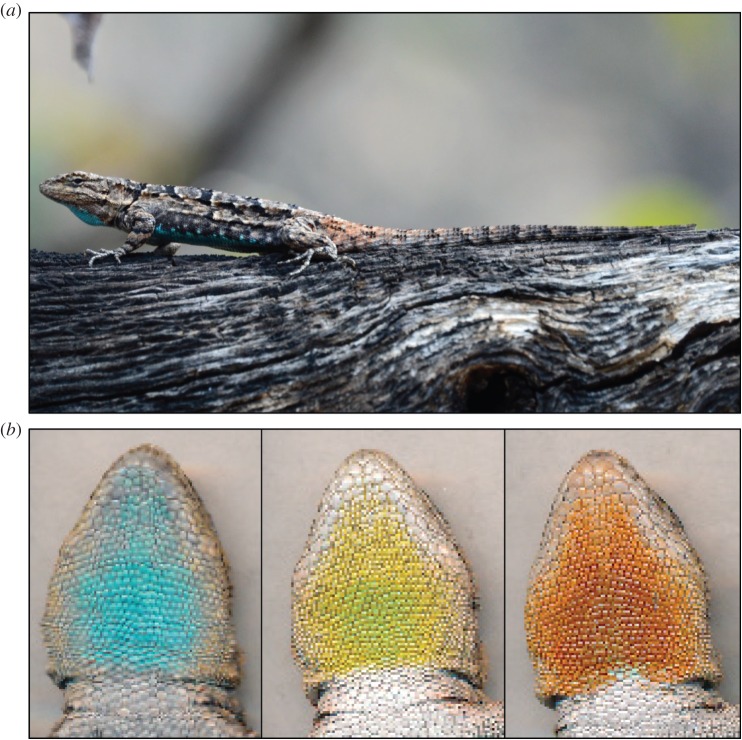
Adult male *Urosaurus ornatus* at our high-burn site (*a*), and images (ventral scans) of the three male dewlap colour morphs (blue, yellow and orange) used in our study (*b*). Photograph in (*a*) by Cynthia Morris and scans in (*b*) by M.S.L.

The ability to describe a trophic polymorphism as well as understand the mechanisms underlying differences in trophic niche occupancy among morphs requires data on their resource-use patterns across contrasting habitats. That is, do morphs select a unique suite of prey types across habitats independent of changes in prey availability and/or habitat quality between habitats? By contrast, do morphs consume prey types in proportion to their availability at a given site? These patterns in trophic niche variation are not captured by estimating diet properties in a single site or by addressing either habitat or prey selectivity alone. In this study, we adopt an integrative approach that capitalizes on the use of stable isotope methods to test whether male colour morphs of the tree lizard, *Urosaurus ornatus* (figure 1), exhibit trophic niche divergence. Stable isotope methods are useful here, because photosynthetic pathway differences between C_3_ and C_4_ vegetation cause their tissues, and the tissues of any organism relying on them as a food resource (either directly or indirectly), to differ predictably in carbon isotopic composition [20], and variation in a consumer's role in a food web will be reflected in the nitrogen isotopic composition of its tissues [22]. Stable isotope data therefore provide information on the variation in base resources of a food web (carbon; i.e. C_3_ versus C_4_ vegetation) and consumer trophic level (nitrogen), which have been assimilated over timescales varying from weeks to years [23]. As a result, isotopic data represent a long-term integration of diet that is more informative from an evolutionary standpoint than diet differences determined from point estimates (e.g. gut-content analyses).

We sampled *U. ornatus* populations in a semi-arid grassland region of southeastern Arizona. We selected two sites that contrast in habitat structure and C_3_ and C_4_ base-resource availability owing to prescribed burning. Prior to the 1920s, periodic fires ignited by lightning strikes maintained the semi-arid grassland environments. However, prolonged fire suppression resulted in an invasion of the grassland ecosystem by trees and shrubs. Recent management practices reinstated burns to mimic historical processes and reduce recruitment of tree and shrubby vegetation (C_3_) in favour of grass (C_4_) propagation [24]. As a result, sites differing in burn history also vary predictably in structural heterogeneity and patterns of vegetation dominance [11]. This variation between our study sites contributes to differences in microhabitat availability and use by *U. ornatus* morphs, as well as in the diversity of prey types supported at each site [25].

Finally, we also consider whether morphological variation among the colour morphs plays a role in their diet selection. In particular, colour morphs in other lizard species differ in head shape [26,27], which is linked to variation in gape size. Because diet is limited by gape size in a small-bodied lizard-like *U. ornatus* [28], variation in head shape among the morphs may also correspond with variation in their diet. The between-site habitat differences, coupled with potential variation in morph head shape, provide us a unique opportunity to test whether male *U. ornatus* colour morphs that exhibit alternative mating tactics also diverge in trophic niche occupancy.

## 2. Material and methods

Tree lizards (*U. ornatus*) at our site are arboreal and establish home ranges in trees within semi-arid grasslands, including oaks (*Quercus* sp.) and mesquite (*Prosopis* sp.). Male *U. ornatus* from many populations are characterized by a discrete polymorphism in dewlap coloration, which is associated with alternative mating tactics [29]. Specifically, blue males are aggressive and territorial [29], yellow males appear to exhibit a satellite behavioural tactic (electronic supplementary material, table S1 and figure S1; see also [11]), and orange males are nomadic [30,31]. We quantified the trophic niche of each morph using stable isotope analysis by sampling lizard activity and resource availability in an eight week period during the breeding season (June–August 2009) at the Appleton-Whittell Research Ranch in Santa Cruz County, Arizona (31°35′ N, 110°30′ W). We obtained data from lizards at two sites that differ in burn history: a low-burn (LB) site had one fire event in 2002, and a second high-burn (HB) site had two fires (1980 and 2002). Aside from differences in burn history, the sites have similar elevation, aspect, slope and soil characteristics. Moreover, all three male *U. ornatus* colour morphs occur in syntopy within these sites.

### 2.1. Lizard capture protocol and resource availability and use

Male *U. ornatus* were collected from within the LB (*n* = 22) and HB (*n* = 18) sites via noose-pole, and all capture points were georeferenced using a portable GPS unit. Lizard surveys were conducted daily between 08.00 and 18.00 h to ensure sampling accounted for the daily activity period of lizards at the study sites. We evaluated whether male morphs selected microhabitat characteristics that differed from the available habitat by sampling within the home range of each lizard (=resource use) and also at non-capture points (=resource availability) established along a grid within each site. These capture points are representative of the resources used by *U. ornatus*, because at this point the territories and spacing patterns among males have stabilized at our sites [11]. Each grid had a total of 32 non-capture sampling points within it that were positioned at 25 m intervals from one another to span the range of potential microhabitats available to the lizards at each site. Preliminary exploration of the microhabitat data collected at these points supports that they are representative of the microhabitats available for *U. ornatus*: they accounted for the full range of available microhabitat types at each site (i.e. 0–100% grass cover, and thus 100–0% shrub per tree cover as well). At each point (use or available), we sampled within 100 m^2^ (approx. 5.6 m radius) plots (centred on the point of capture or an availability point, respectively), because the mean territory of *U. ornatus* is less than this size [32,33].

Four 1 m^2^ frames were placed randomly within each 100 m^2^ sampling plot. Within each frame, we estimated the per cent cover of vegetation (grass, forb, shrub and tree) and structural features (bare ground, rocks (more than 2 cm diameter), woody debris and leaf litter). All cover variables were estimated to the nearest 5%. The averages of the coverage data based on the four frames from each plot were retained for analysis. We also recorded distance to the nearest tree from the centre of the plot (nearest 0.1 m). We collected vegetation samples (leaf, stems and flowers) within each plot for isotopic analysis and stored these samples in separate plastic bags within a freezer bag.

We determined the available arthropod diversity at each site by systematically sweep netting each study site for 4 h per day over a 2 day period at each site (thus eight total survey hours per site). We netted for approximately 2 h during the morning (*ca* 09.00–11.00 h) and 2 h during the afternoon (*ca* 13.00–15.00 h) for each sweep-net day. This method captured representative arthropods from every major group identified by Asplund [34], including some groups not included in that study that may be difficult to sample (e.g. Orthoptera [35]). We placed each arthropod into a separate 2 ml plastic vial and immediately placed the samples on ice. We identified all specimens to family prior to isotopic analysis. A total of 25 and 30 arthropod families were identified at the HB and LB sites, respectively.

### 2.2. Morphology

For each male lizard captured, we recorded the colour pattern of the dewlap, mass, as well as snout–vent length (SVL), head width (HW) and jaw length (JL). Mass was measured to the nearest 0.1 g using a spring scale, and SVL, HW and JL were measured to the nearest 0.1 mm using callipers. We toe-clipped all males to prevent recapture (two toes per male). The claws from these toes were retained for stable isotope analysis prior to release at their capture sites.

### 2.3. Stable isotope analysis

Patterns in trophic polymorphisms are often based on point estimates derived from prey items identified by stomach flushing or behavioural observations. Such estimates represent a short-term characterization of prey consumption by an organism over a period of hours. We suggest that trophic variation characterized using stable isotope methods represents a more inclusive estimate of individual diet [36,37].

All tissue samples (vegetation, arthropod and lizard claw) were stored in a freezer at −20°C until the end of the study. We then oven-dried samples at 60°C for 48 h, and then ground and homogenized the dried samples separately. We weighed and transferred all dried samples into separate 5 × 8 mm tin capsules (Costech Inc., Valencia, CA). We pooled some arthropod samples by family, because of their small mass, to generate enough tissue for isotopic processing (e.g. ants, leafhoppers). These samples were then sent to the University of California Davis Stable Isotope Facility (http://stableisotopefacility.ucdavis.edu) for analysis of *δ*^13^C and *δ*^15^N values using a PDZ Europa ANCA-GSL elemental analyser interfaced to a PDZ Europa 20–20 isotope ratio mass spectrometer (Sercon Ltd., Crewe, Cheshire, UK). The error deviation for *δ*^13^C was 0.2‰ and 0.3‰ for *δ*^15^N. All isotopic values generated by this analysis are expressed in standard per-mil delta (*δ*) notation,

$$X‰=\left(\frac{R_{\mathrm{sample}}}{R_{\mathrm{standard}}}-1\right)\times 1000‰,$$

where *X* = *δ*^13^C or *δ*^15^N and *R* = ^13^C/^12^C or ^15^N/^14^N, respectively. Our experimental design allowed calculation of *δ*^15^N and *δ*^13^C values from *n* ≥ 3 replicate samples of each vegetation type and arthropod consumer type per site.

### 2.4. Statistical analysis

We conducted all statistical analyses in R v. 2.15 [38]. We compared vegetation and structural cover data by site (availability points only) with a non-parametric multivariate analysis of variance (MANOVA). We ran a separate non-parametric MANOVA to test whether vegetation and structural cover data differ by type (use (capture point) versus availability) and the interaction between site and type (i.e. if morphs shift in habitat use depending on changes in availability of structural or vegetation resources). To test whether morphs differ in microhabitat use between the two sites, we used a non-parametric MANOVA with the resource use data as the response variable matrix with morph and site as factors. Our analyses for each model were based on 4000 permutations, with limited randomizations to within-sites only, and response variable distance matrices derived from a Bray–Curtis dissimilarity metric (function ‘adonis’ in the vegan package) [39]. We used separate non-metric multidimensional scaling (NMDS) ordinations to explore differences between the characteristics of used and available microhabitats at each site. To determine whether any variable(s) explained variation in each NMDS ordination, we evaluated the correlation between each input variable and the first two axes generated by the ordination. Because there are no post hoc tests for permutation procedures such as ‘adonis’, we outlined an ellipse representing the 95% confidence limit of the centroid for each factor group in each ordination plot. Significant differences between pairs of factor groups occur if these ellipses do not overlap.

We applied Sorenson's D coefficient to presence–absence data of arthropod families to determine the dissimilarity between sites in arthropod composition [40]. The value of *D* ranges from 0 to 1, with greater values indicating greater dissimilarity. We compared arthropod diversity (number of families) between the sites using chi-square statistics.

We used a multinomial test to compare morph frequencies between the sites. We assessed head shape variation among the male morphs at each site using a multivariate analysis of covariance (MANCOVA) with morph and site as fixed factors, SVL as a covariate, and HW and JL as response variables. We analysed the magnitude of the spatial segregation among morphs at each site using separate MANCOVAs with morph as a fixed factor, distance to the nearest tree as a covariate, and normalized latitude and longitude of each lizard capture point as response variables. Here, normalization refers to centring of the coordinate data to a mean of zero and unit standard deviation. We included tree distance as a covariate, because the distribution of this species may be influenced by tree density. Prior to running these MANCOVAs, we ensured homogeneity of all regression slopes (morphology, both interactions *p* > 0.4; spatial segregation, interaction *p* > 0.7).

We noted that shrubs and trees exhibited high overlap in isotopic delta-space, but significant differences occurred between these types and grasses and forbs. We divided the individual plants sampled at each site (*n* = 85 and *n* = 45 for the LB and HB sites, respectively) into three types: grass, forb or shrub (including trees). We used a MANOVA to test for differences in the stable isotope values of vegetation with site and vegetation type as factors and *δ*^13^C and *δ*^15^N values as the response variables. Following Gratton & Denno [41], we divided arthropods into one of four consumer types: C_3_ herbivores (some grasshoppers, leafhoppers and stick insects), C_4_ herbivores (most grasshoppers), non-spider predators (some ants, beetles and true bugs), and spiders. We tested for differences in the carbon and nitrogen stable isotope values of each of these consumer types between the two sites using a MANOVA with site and consumer type as factors and *δ*^13^C and *δ*^15^N values as the response variables.

We estimated trophic linkages between vegetation, arthropods and lizard colour morphs at each site using separate mixing models implemented with the function ‘siarmcmcdirichletv4’ in the SIAR package [42]. This function uses a Bayesian approach to estimate the possible range of contributions of each resource to each consumer based on their isotopic values and any expected change between them (i.e. discrimination factor, [43]). The mean likelihood of contribution between each resource and a consumer can therefore be retained as an estimate of the linkage between a predator and its prey. We validated the use of carbon and nitrogen isotopes as estimators of the trophic niche for *U. ornatus* from claw tissue in another experiment [44]. We used the discrimination factors obtained from that experiment (Δ^13^C = 1.2 ± 0.4‰ and Δ^15^N = 0.7 ± 0.3‰) as lizard parameters in our mixing models. We used published discrimination factors for the four arthropod consumer types in these models (table 1).

**Table 1.** Trophic inputs and discrimination factors for four mixing models we implemented using the SIAR statistical package [42] in R. (Discrimination factors (Δ) are estimates of the expected change in stable isotope values between resources and a consumer type [43]. Sources for these data are provided in the last column. Because some studies did not provide a standard deviation (SD), we used the maximum SD reported among all sources we analysed (0.4) as a conservative estimate of SD (indicated by an asterisk).)

			Δ^13^C (‰)		Δ^15^N (‰)		
mixing model	resource end-members	consumer end-member(s)	mean	s.d.	mean	s.d.	source
primary arthropod consumers (C_3_ and C_4_ herbivores)	grass, forb and shrub vegetation	C_3_ herbivores, C_4_ herbivores	3.7, 0.7	0.3, 0.2	3.1, 7.8	0.3, 0.2	Webb *et al.* [45]
secondary arthropod consumers (non-spider predators)	C_3_ herbivores, C_4_ herbivores	non-spider predators	−0.2	0.4*	2.9	0.4*	Ostrom *et al.* [46]
tertiary arthropod consumers (spiders)	C_3_ herbivores, C_4_ herbivores, non-spider predators	spiders	0	0.4*	1.5	0.4*	Gratton & Denno [41]
lizards (*Urosaurus ornatus*)	all arthropod types	lizards	1.2	0.4	0.7	0.3	Lattanzio & Miles [44]

Prior to analysing our lizard diet isotopic data, we estimated the relationship between lizard body condition (residuals from a regression of mass versus SVL, g) and *δ*^15^N values, because both prey selection and diet assimilation can be affected by an organisms' physiological condition [47,48]. We detected no significant relationship between body condition and *δ*^15^N values at either site (LB site, *r* = −0.37, *t*_20_ = −1.8, *p* = 0.094; HB site, *r* = 0.14, *t*_16_ = 0.6, *p* = 0.58). We tested for differences in the trophic niche of each morph using a MANCOVA containing site and morph as fixed factors, SVL as a covariate, and *δ*^13^C and *δ*^15^N values as response variables. We detected no significant interaction between either site or morph and SVL in this model (both *p* > 0.7). We used correlation tests to explore relationships between head shape and diet (isotopic nitrogen data). Finally, we only report Bonferroni-adjusted *p*-values (*p*_adj_) for significant post hoc pairwise comparisons.

## 3. Results

### 3.1. Resource availability and use

The LB and HB sites differed in habitat structure and vegetation cover (adonis, *F*_1,62_ = 10.27, *p* = 0.001; electronic supplementary material, table S2). The LB and HB sites were characterized by variation in structural features of the habitat (NMDS, axis 1: leaf litter, *r* = 0.9, *p* < 0.001; tree distances, *r* = −0.5, *p* < 0.001; rocks, *r* = −0.56, *p* < 0.001; bare ground, *r* = −0.51, *p* < 0.001) and vegetation cover (NMDS, axis 2: grass, *r* = 0.94, *p* < 0.001; forbs, *r* = −0.57, *p* < 0.001). The HB site had higher grass and rock cover, more leaf litter cover, but also had less bare ground cover and fewer forbs than the LB site (electronic supplementary material, figure S2). The structural habitat and vegetation cover at male capture points differed from resource availability points (*F*_1,100_ = 25.15, *p* = 0.001). We detected an interaction between site and plot type (site × plot type, *F*_2,100_ = 6.11, *p* = 0.001). Specifically, the difference in habitat characteristics between lizard capture localities and availability points was greater at the HB site (electronic supplementary material, figure S3). Male microhabitats were characterized by higher tree density, greater amounts of leaf litter, fewer rocks and greater forb cover than resource availability points (electronic supplementary material, figure S3). Male morphs used microhabitats with similar characteristics at both sites (site, *F*_1,34_ = 2.4, *p* = 0.75; morph, *F*_2,34_ = 0.35, *p* = 0.92; site × morph, *F*_2,34_ = 0.9, *p* = 0.4).

We collected a higher diversity of arthropods at the family level at the LB site (by family: Sorenson's D = 0.83; $\chi_{1}^{2}$ = 7.57, *p* = 0.01). We captured a higher diversity of non-spider predators and spiders at the LB compared with the HB site (14 versus 8 and 5 versus 3 families identified, respectively).

### 3.2. Isotopic variation in available resources and prey

**Figure 2.**
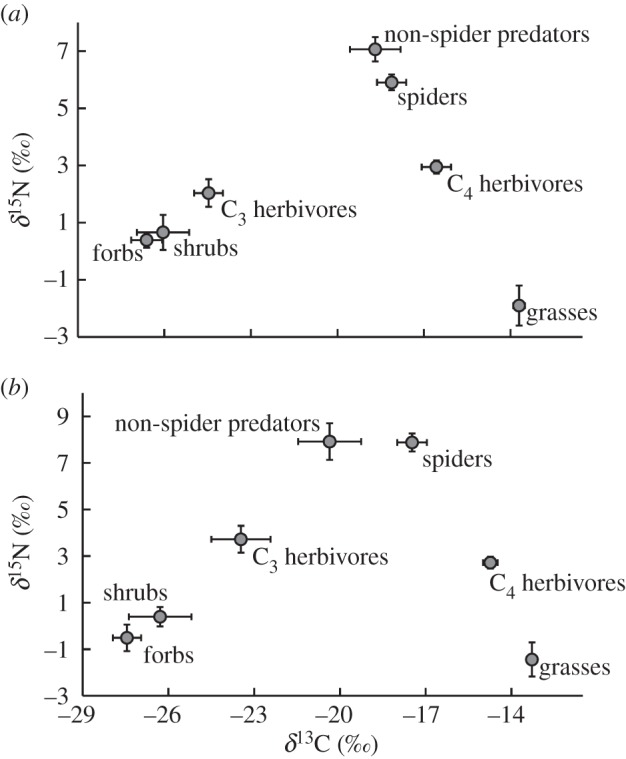
Vegetation (grass, forb and shrub) and arthropod (C_4_ and C_3_ herbivores, non-spider predators and spiders) *δ*^13^C and *δ*^15^N values at the low-burn (*a*) and high-burn (*b*) site. These vegetation and arthropod consumer types were entered into isotopic mixing models to quantify trophic linkages for *Urosaurus ornatus*. Vegetation (grass, shrub or forb) (*n* ≥ 11 each group) and arthropod consumer (C_4_ herbivores, C_3_ herbivores, non-spider predators and spiders) (*n* ≥ 6 each group) types are defined in the Material and methods. Points represent mean values and bars are ± 1 standard error.

Overall, isotopic carbon signatures of C_4_ (grasses, mean = −13.5 ± 0.7‰) differed from C_3_ (forbs, mean = −27.5 ± 1.6‰; shrubs, mean = −26.3 ± 4‰). The *δ*^13^C and *δ*^15^N values of vegetation differed by site and type only (site, *F*_1,124_ = 5.98, *p* = 0.003; vegetation type, *F*_2,124_ = 34.83, *p* < 0.001; site × vegetation type, *F*_2,124_ = 0.88, *p* = 0.48). The vegetation at the LB site is characterized by a depletion in *δ*^13^C and *δ*^15^N values in relation to the HB site (figure 2 and electronic supplementary material, table S2). In addition, grasses are enriched in *δ*^13^C and depleted in *δ*^15^N values compared with grasses and forbs (all *p* < 0.001). The differences in isotopic values between the sites is supported by the higher proportional cover of C_3_ vegetation at the LB site (forb, $\chi_{1}^{2}$ = 12.48, *p* < 0.001; shrub, $\chi_{1}^{2}$ = 1.93, *p* = 0.17) compared with greater C_4_ vegetation cover at the HB site (grass, $\chi_{1}^{2}$ = 11.3, *p* < 0.001).

The *δ*^13^C and *δ*^15^N values of arthropods differed by consumer type but not by site (site, *F*_1,143_ = 1.79, *p* = 0.17; consumer type, *F*_3,143_ = 46.57, *p* < 0.001; figure 2). Moreover, although most arthropods were enriched in both *δ*^13^C and *δ*^15^N values in the HB site, this trend was not significant overall (site × consumer type, *F*_3,143_ = 2.06, *p* = 0.06; table 2, and electronic supplementary material, table S2).

**Table 2.** Relationships of *δ*^15^N and *δ*^13^C values of each arthropod consumer type identified at the two sites. (Sites are delineated by historical disturbance frequency: low- or high-burn. *p*-values are from post hoc pairwise comparisons from a MANOVA comparing stable isotope values of each arthropod type by location (low- or high-burn site, respectively; see Material and methods). Isotopic delta-values are presented as mean ± 1 standard error. Significant *p-*values are italicized.)

	low-burn site		high-burn site		N	C
arthropod consumer type	*δ*^15^N	*δ*^13^C	*δ*^15^N	*δ*^13^C	*p-*value	*p-*value
C_4_ herbivores	3 ± 0.2	−16.6 ± 0.5	2.7 ± 0.3	−14.7 ± 0.3	0.53	*0.014*
C_3_ herbivores	2 ± 0.5	−24.5 ± 0.5	3.7 ± 0.6	−23.5 ± 1	*0.031*	0.37
non-spider predators	7.1 ± 0.4	−18.7 ± 0.9	7.9 ± 0.8	−20.4 ± 1.1	0.3	0.25
spiders	5.9 ± 0.3	−18.1 ± 0.5	7.9 ± 0.4	−17.5 ± 0.5	<*0.001*	0.45

### 3.3. *Urosaurus ornatus* colour morph frequencies, morphology and spatial segregation

Morph frequencies differed between the sites ($\chi_{2}^{2}$ = 9.32, *p* = 0.01), with blue males less common at the HB site ([Y, O, B]: LB site, 8, 5, 9; HB site: 9, 6, 3). Male lizards were larger in body size at the HB site (HB, 49 ± 0.5 mm; LB, 46.9 ± 0.5 mm; electronic supplementary material, table S2). Head shape differed by morph but not by site (site, *F*_1,33_ = 3.14, *p* = 0.057; morph, *F*_2,33_ = 9.31, *p* < 0.001). Blue morph males had longer jaws than either yellow or orange males (yellow males, *p*_adj_ < 0.001; orange males, *p*_adj_ = 0.04), and yellow males had longer jaws than orange males (*p*_adj_ < 0.001; see also the electronic supplementary material, table S2). Moreover, the patterns of head shape variation among the morphs also differed by site (site × morph, *F*_2,33_ = 6.57, *p* < 0.001). The jaws of yellow males at the HB site were longer than those of yellow males at the LB site (*p*_adj_ = 0.003), but similar in size to those of blue males at the HB site (*p*_adj_ = 0.28). There was no effect of body size on head shape (*F*_1,33_ = 2.41, *p* = 0.11). Finally, we detected only spatial segregation among male morphs at the LB site (LB site, *F*_2,18_ = 4.17, *p* = 0.007; HB site, *F*_2,14_ = 0.6, *p* = 0.67).

### 3.4. Trophic niche variation

**Figure 3.**
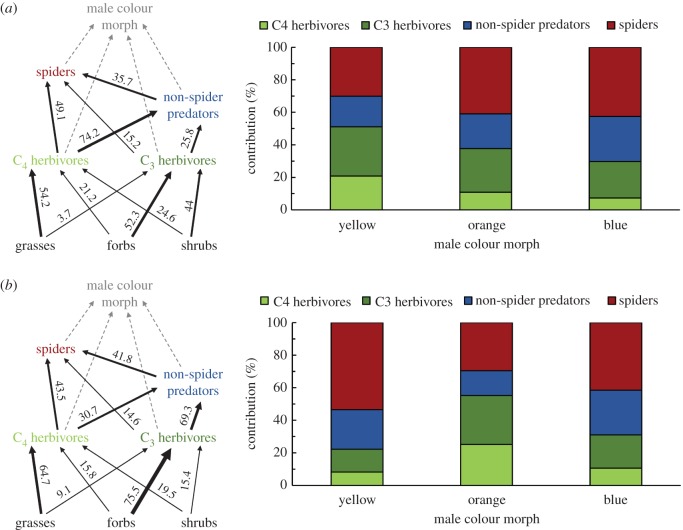
Results of Bayesian mixing models of contributions (%) of each resource to each consumer at the (*a*) low-burn and (*b*) high-burn sites. Arthropod mixing models are illustrated as paths (arrows), weighted by the contribution of each resource to each consumer. Estimated per cent contributions are also provided along each path. Grey broken paths denote the general mixing model used to estimate the per cent contributions of the four arthropod groups to male tree lizard (*Urosaurus ornatus*) diets. Estimated contributions of these four consumer types to each male colour morph (yellow, orange or blue) are provided as a stacked bar graph for each site. Section colours of each bar correspond to the text colour of that consumer type in the arthropod mixing models. Arthropod consumer types are defined in the Materials and methods, and model parameters are provided in table 1. Mixing models were implemented in R using the function ‘siarmcmcdirichletv4’ within the package SIAR [42] (*n* ≥ 3 for each group).

Overall, C_3_ herbivores in the LB site exploited shrubs and forbs in similar proportions (44% and 52%, respectively), but primarily exploited forbs at the HB site (figure 3). C_4_ herbivores were more consistent in resource use across sites (more than 54% reliance on grasses at both sites). Non-spider predators in the HB site preferred C_3_ herbivores over C_4_ herbivores (69% versus 31%), which contrasts with their preference in the LB for C_4_ herbivores overall (74%). Spiders maintained consistent diets across sites and favoured C_4_ herbivores and non-spider predators over C_3_ herbivores (approx. 15% reliance on C_3_ herbivores at both sites).

The trophic niche of blue and orange tree lizards at the LB site was characterized by a higher consumption of spiders (43% and 41%, respectively; figure 3). Yellow tree lizards showed no preference toward a specific prey type and consumed all four prey types at near-equal frequencies (approx. 19–30% for each source). At the HB site, trophic niche relationships among the morphs shifted such that yellow males converged with blue males in trophic niche, favouring non-spider predators and spiders over other sources (more than 24% for each type, 69–77.8% total). By contrast, orange males primarily consumed herbivorous arthropods at this site (55% total use).

**Figure 4.**
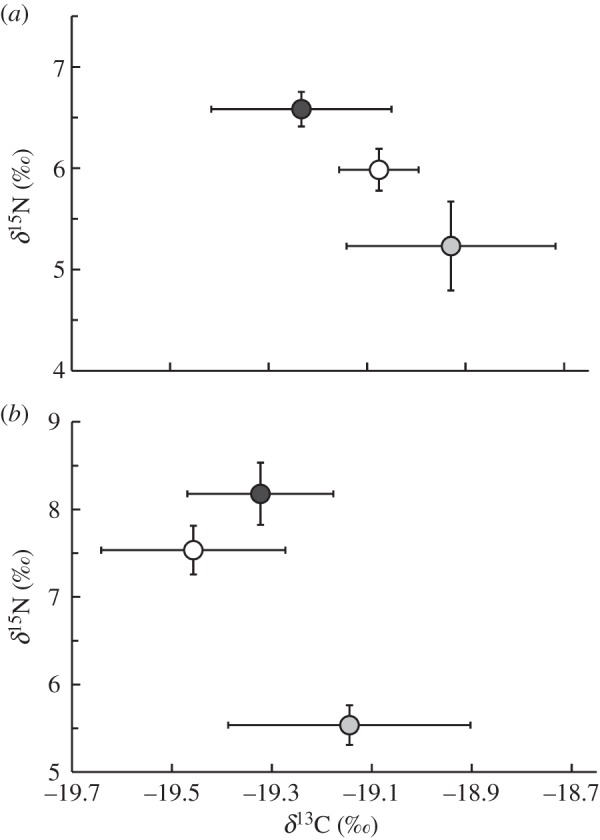
Trophic niche differences among male *Urosaurus ornatus* colour morphs at the (*a*) low-burn and (*b*) high-burn sites. Points are mean δ^13^C and δ^15^N values and are shaded by dewlap colour: yellow (light grey), orange (grey) or blue (dark grey). Bars are ± 1 standard error (*n* ≥ 3 for each group).

The diet of lizards captured at the HB site is enriched in both *δ*^15^N and *δ*^13^C values compared with the diet of lizards captured at the LB (figure 4 and electronic supplementary material, table S2). Morphs differed in diet between the sites (site, *F*_1,33_ = 8.56, *p* = 0.001; morph: *F*_2,33_ = 6.79, *p* < 0.001; site × morph: *F*_2,33_ = 1.67, *p* = 0.17). There was no effect of body size on diet (*F*_1,33_ = 1.61, *p* = 0.21). The lack of interaction between site and morph is because of the high similarity of prey *δ*^13^C values between both sites (i.e. figure 2). Yellow and blue males consumed prey from higher trophic levels than orange males (both *p*_adj_ < 0.001), but yellow males in the HB site foraged at a higher trophic level than yellow males in the LB site (*p*_adj_ = 0.001). In terms of head shape, JL positively correlated with diet (isotopic nitrogen data) in our overall sample (i.e. regardless of morph, *r* = 0.48, *t*_38_ = 3.37, *p* = 0.002). Likewise, the shift in diet by yellow males at the HB site corresponded with their variation in head shape (*r* = 0.58, *t*_15_ = 2.79, *p* = 0.014).

## 4. Discussion

Our results revealed a trophic niche polymorphism in male tree lizards that coincided with the colour morphs. The patterns emerging from our analysis support the hypothesis that habitat variation contributes to the relative spatial segregation of morphs and the spatial variation in diversity of available prey that shape trophic divergence. Variation in diet among morphs has long been considered to play a key role in the speciation process [14], especially if morphs also differ in reproductive behaviour [49]. In another study, we provided evidence that male *U. ornatus* colour morphs may feed at different trophic levels and exhibit greater spatial overlap in resource-limited habitats [11]. Our current findings expand those patterns by demonstrating that morphs also differ in their degree of diet specialization and probably occupy distinct trophic niches. To the best of our knowledge, ours is only the second study to quantify variation in diet among morphs in a colour polymorphic species where morphs diverge in reproductive behaviour [18], and the first to provide evidence that colour morphs may also diverge in foraging tactics (see below).

Trophic polymorphisms are expected to evolve via one of two avenues. Trophic divergence may evolve in sympatry when morphs vary in their ability to exploit different prey types [13]. In *Podarcis melisellensis* [6], dietary variation among morphs in sympatry is attributed to morph differences in bite force and morphology (head and body size). Larger orange coloured males have a higher bite force and, consequently, their diet includes a higher proportion of hard prey in their gut than white or yellow morph males [6]. Alternatively, exploitation of different microhabitats by morphs favours divergence in trophic niche through local adaptation [17,50]. In Italian wall lizards (*Podarcis muralis*), morph-specific differences in diet based on analysis of faecal pellets are attributed to potential spatial segregation of morphs within a shared habitat [50], given that the species is assumed to be a generalist, opportunistic forager [28]. However, these two hypotheses are limited in scope: whereas the first hypothesis ignores the influences of habitat variation and prey diversity on diet, the second hypothesis ignores variation in diet selectivity among discrete morphs.

Our findings support a previously unconsidered mechanism for the generation of a trophic polymorphism, namely that microhabitat segregation arising from behavioural interactions among males differing in mating tactics exposes individuals to different resource bases. Including the role of behaviour and differences in habitat quality provides a mechanism to explain the evolution of a trophic polymorphism in species characterized by alternative mating tactics. For example, the degree of dietary specialization among *P. melisellensis* morphs (at least, in terms of proportion of hard and soft prey items, see [6]), would probably depend on vegetation and prey diversity in the area: in more diverse habitats, dietary overlap should be favoured, regardless of morph differences in size or behaviour [51]. This mechanism also allows morphs to vary in their degree of trophic specialization (e.g. generalist or specialist) as they do in mating behaviour and other physiological traits [2–4,18,29,52–54]. Our hypothesis synthesizes and expands on the mechanisms proposed by Skúlason & Smith [13] and Karpestam & Forsman [17] to explain the evolution of a trophic polymorphism. However, explicit comparisons of prey availability, habitat variation and morph diets among sites occupied by other polymorphic species [50] are needed to enhance our understanding of the mechanisms contributing to the evolution of trophic polymorphisms in nature.

The extent of the trophic polymorphism depends on variation in habitat quality. For example, consistent dietary differences among morphs in sympatry across different habitats should only be expected to arise if they are an outcome of similar mechanisms operating within each morph, such as selectivity for specific prey types, regardless of shifts in prey availability among habitats. However, if morphs differ in their mechanisms of prey selection, as they do in mating behaviour, then habitat variation should influence their trophic niche differences. In high-quality habitats, dietary overlap among morphs may arise, because prey and microhabitat resources are abundant. By contrast, poorer-quality habitats (e.g. burned areas) with limited resources would instead favour ecological segregation of morphs based on their differences in aggressive behaviour, potentially enhancing their trophic niche differences. These considerations emphasize the need for trophic niche data across contrasting habitats in order to draw conclusions regarding the nature of a potential trophic polymorphism.

The patterns of diet and morphological divergence among the morphs at our study sites coincide with their behavioural traits. Among males inhabiting the LB site, blue and orange morphs have a higher percentage of predatory arthropods in their diet, whereas yellow males consume all arthropod types at a similar frequency. Greater tree and shrub cover, and higher predatory arthropod diversity, is common at sites that are burned infrequently [25,55]. Greater habitat complexity combined with the high availability of prey resources allows morphs to spatially segregate and results in unique access to diverse resources. By contrast, the HB site had fewer trees and shrubs but a higher coverage of grasses, and male morphs exhibited greater spatial overlap. We found greater trophic niche divergence among the morphs at this site. The diet of blue and yellow males consisted primarily of predatory arthropods. Previous studies have shown that orange males are nomadic [29,30], and their generalist diet reflects a pattern that tracks prey availability at this site.

The divergent patterns emerging from these two sites add support for the importance of habitat heterogeneity in the generation and maintenance of trophic niche variation within a population [56,57]. However, the observed trophic patterns in *U. ornatus* are also contingent on behavioural asymmetries among the male morphs, as our mixing model results support that morphs also differ in their degree of diet specialization. Dominant blue males usurp high-quality patches and exhibit the greatest dietary specialization at both sites. Orange males are nomadic and may encounter a broader range of prey types as they move through the habitat. Our isotopic data for orange males suggest they are trophic generalists as their diet tracks changes in prey availability between the sites. By contrast, yellow males exhibit a plastic dietary niche that involves a shift in diet between sites that is unrelated to changes in prey availability. In addition, their differences in resource use were also linked to head shape morphology: blue males had longer heads than the other morphs, and yellow males in the HB site had longer heads than yellow males in the LB site. The shift in diet by yellow males is probably associated with their satellite behavioural tactic (e.g. electronic supplementary material, table S1 and figure S1), coupled with the decrease in frequency of dominant blue males at the HB site (see also [11]). In addition, the elongation of yellow male jaws coincides with their dietary overlap with blue males at the HB site, supporting a functional linkage between head shape morphology and diet in this morph [50]. In another study on a *U. ornatus* population in northcentral Arizona, head shape does not differ among morphs, although we should note that in general, blue and yellow males had longer jaws than orange males in that study [58]. Studies of other colour polymorphic species, however, support that colour morphs may differ in head shape [26,27]. Thus, because head shape influences diet selection in small-bodied lizards [28], morph-specific variation in head shape may be associated with exploitation of different dietary resources. Our current findings support this prediction. Moreover, plasticity in head shape may allow yellow males in particular to usurp novel dietary niches when they become available. Altogether, our results suggest that *U. ornatus* male colour morphs also exhibit distinct ecological tactics in addition to mating behaviours, and that a trophic polymorphism may also enhance morphological diversity in resource-limited environments.

## 5. Conclusion

Studies of the factors maintaining colour polymorphism within a species have generally focused on the fitness consequences of individual variation in mating behaviour, with scant attention towards the potential ecological consequences of differences in morph behaviour. By contrast, trophic polymorphisms are attributed to divergent natural selection associated with either microhabitat or prey selectivity alone. The results of our study suggest an alternative mechanism for the generation of a trophic polymorphism: diet differences among morphs may be a consequence of both socially mediated sexual selection and natural selection mechanisms. The differences in diet and foraging tactics we document in *U. ornatus* are coincident with their colour polymorphism and associated with increased morphological diversity (in terms of head shape). Collectively, these findings support a role for trophic polymorphism in enhancing rates of ecological divergence in colour polymorphic taxa [14,49].

## Supplementary Material

Table S1. Results of analysis of yellow male U. ornatus satellite behaviour. Table S2 - Summary of vegetation, arthropod, and U. ornatus datasets used in this study. Figure S1 - Variation in spatial proximity (in metres) of yellow morph U. ornatus to either blue or orange males from a nearby population. Figure S2 - Non-metric multidimensional scaling ordination showing microhabitat variation between the two study sites. Figure S3 - Non-metric multidimensional scaling ordination showing microhabitat variation between lizard capture and gridded availability (non-capture) points in each site.

## Supplementary Material

Raw Data S1 - Raw data (XLSX).

## Data accessibility

Supporting materials (including raw data) for this article are included in the accompanying electronic supplementary material.
