# Supplementary material for: Trophic niche divergence among colour morphs that exhibit alternative mating tactics
Source: R Soc Open Sci. 2016 Apr 13;3(4):150531. doi: 10.1098/rsos.150531 (PMC4852626; doi:10.1098/rsos.150531)

# Electronic Supplementary Material for

# Title: Trophic niche divergence among colour morphs that exhibit alternative mating tactics

**Authors:** Matthew S. Lattanzio and Donald B. Miles

# Journal: Royal Society Open Science

# Supplemental Tables and Figures

**Table S1**. Results of linear mixed-effects models testing whether yellow male *U. ornatus* exhibit a satellite behavioural tactic and cluster in close proximity to blue male territories, or are nomadic like orange males. If yellow males are satellite to blue male territories, we predict that they should spatially cluster around blue males. In contrast, nomadic males should be equidistant to the other two morphs. We used as a response variable the distance of a yellow male (in metres) to the nearest blue or orange male (regardless of its individual identity) based on the georeferenced capture points of all males collected during June-July of 2013 (blue males [n=16], orange males [n=3], yellow males [n=10]) and 2014 (blue males [n=13], orange males [n=3], yellow males [n=20]) ~2 km from our current study sites. We used Akaike Information Criteria (AIC) to evaluate the goodness-of-fit among candidate models that differed in how they included of two fixed effects: year, and nearest male morph (orange or blue, respectively). All models included individual id as a random effect. Our best-supported model (i.e., model with the lowest AIC) included the main effects of year, neighbour morph, and their interaction (Model ‘LM_i_’ below) on neighbour distance. These results verify the satellite status of yellow males. Satellite males are closer to blue than they are to orange males, regardless of year (post-hoc pairwise tests using α-corrected *P*-values, all *P* <0.001; see also Figure S1). We omitted the distance between neighbouring yellow males in this analysis because our goal was to test for heteromorphic variation in spatial clustering by this morph to elucidate their behavioural tactic (if any) with respect to the other two morphs. Inclusion of all three morph neighbour possibilities in a separate analysis of neighbour morph frequencies however yields similar findings: yellow males prefer blue neighbours (Chi-Square test: *χ*^2^ = 6.2, df = 2, *P* = 0.045).

| Model name | Formula | AIC | ΔAIC | *w*_i_(AIC) |
| --- | --- | --- | --- | --- |
| LM_0_ | ~intercept | 684.3 | 30.8 | 0 |
| LM_y_ | ~year | 669.5 | 16 | 0 |
| LM_n_ | ~neighbour | 674.9 | 21.4 | 0 |
| LM_yn_ | ~year + neighbour | 659.8 | 6.3 | 0.04 |
| LM_i_ | ~year + neighbour + year × neighbour | 653.5 | 0 | 0.96 |

**Table S2**. Summary of vegetation, arthropod, and *Urosaurus ornatus* datasets used in this study. Vegetation variables are measured as percent cover (%) with the exception of tree distances (meters). Isotopic (*δ*^13^C and *δ*^15^N) values are expressed as per-mil (‰). Vegetation data are summarized by plot type (gridded availability point or capture point), as well as by male throat color (yellow, orange, or blue). Here, ‘Capture points’ refers to vegetation cover data averaged across the capture points of all three male morphs. Vegetation sampling protocol, vegetation types, and arthropod consumer types are described in Material and Methods. Morphological data for each lizard morph include snout-vent length (SVL), head width (HW), and jaw length (JL), in mm, and mass, in g. Values in each cell are mean (standard error) (n ≥ 3 each group).

| (a) LB Site |  | Variable | | | | | | | |
| --- | --- | --- | --- | --- | --- | --- | --- | --- | --- |
| *Vegetation Cover (%)* | | Grass | Forb | Shrub | bare ground | Rock | Leaf Litter | Woody Debris | Tree distance (m) |
| Availability points |  | 29.9 (4.2) | 16.6 (3.8) | 2.5 (0.7) | 27.3 (2.3) | 15.2 (2.5) | 10.2 (2.6) | 0.8 (0.4) | 14.1 (1.1) |
| Capture points |  | 18.9 (2.2) | 3 (0.7) | 2.7 (0.8) | 26 (4.6) | 16.1 (4.2) | 28.1 (5.4) | 9.1 (1.8) | 2.2 (0.5) |
| Male Morph | Yellow | 19.4 (3.5) | 1.5 (0.6) | 2.9 (1.6) | 22.4 (7.7) | 14.8 (7.7) | 31.3 (9.9) | 10.3 (3) | 2.8 (1.4) |
|  | Orange | 21.7 (4.5) | 3.6 (0.8) | 2.4 (1.1) | 33.7 (9.4) | 16 (6.4) | 17.5 (9) | 7.8 (3.1) | 2.4 (0.3) |
|  | Blue | 16.6 (4) | 3.9 (1.6) | 2.7 (1.3) | 24.1 (4) | 17.4 (7.7) | 32.4 (9.3) | 8.9 (3.3) | 1.4 (0.4) |
| *Isotopic and Morphological Data* | | *δ*^13^C (‰) | *δ*^15^N (‰) | SVL (mm) | HW (mm) | JL (mm) | Mass (g) |  |  |
| Vegetation |  |  |  |  |  |  |  |  |  |
|  | Grass | -13.7 (0.2) | -1.9 (0.7) | - | - | - | - |  |  |
|  | Forb | -26.6 (0.5) | 0.4 (0.3) | - | - | - | - |  |  |
|  | Shrub | -26 (0.9) | 0.7 (0.6) | - | - | - | - |  |  |
| Arthropods |  |  |  |  |  |  |  |  |  |
|  | C_4_ -Herbivores | -16.6 (0.5) | 3 (0.2) | - | - | - | - |  |  |
|  | C_3_ -Herbivores | -24.5 (0.5) | 2 (0.5) | - | - | - | - |  |  |
|  | Non-spider Predators | -18.7 (0.9) | 7.1 (0.4) | - | - | - | - |  |  |
|  | Spiders | -18.1 (0.5) | 5.9 (0.3) | - | - | - | - |  |  |
| Lizard |  |  |  |  |  |  |  |  |  |
| Male Morph | Yellow | -19.1 (0.1) | 6 (0.2) | 48.2 (0.6) | 8.9 (0.2) | 8.6 (0.1) | 2.6 (0.3) |  |  |
|  | Orange | -18.9 (0.2) | 5.2 (0.4) | 45.3 (1.2) | 9.3 (0.2) | 7.9 (0.1) | 3.2 (0.3) |  |  |
|  | Blue | -19.2 (0.2) | 6.6 (0.2) | 46.7 (0.9) | 9.3 (0.2) | 9.3 (0.1) | 2.8 (0.3) |  |  |
|  |  |  |  |  |  |  |  |  |  |
|  |  |  |  |  |  |  |  |  |  |
| **Table S2 (cont’d)** | |  | | | | | | | |
| (b) HB site |  | variable | | | | | | | |
| *Vegetation cover* (%) | | grass | forb | shrub | bare ground | rock | leaf litter | woody debris | tree distance (m) |
| availability points |  | 48.6 (3.5) | 1.5 (0.7) | 1.3 (0.5) | 30.9 (3.2) | 7.5 (2) | 11.3 (2.7) | 0.8 (0.4) | 6.8 (0.6) |
| capture points |  | 18.2 (3.1) | 0.6 (0.2) | 1.8 (0.7) | 27.5 (4) | 2.6 (1.2) | 45 (5.9) | 10.4 (2.6) | 2.6 (0.4) |
| male morph | yellow | 26 (3.9) | 0.5 (0.3) | 0.7 (0.6) | 26.2 (3) | 1.8 (1.3) | 41 (5.5) | 9.8 (3.1) | 2.7 (0.5) |
|  | orange | 7.5 (2.4) | 0.9 (0.5) | 4.3 (1.7) | 22 (6) | 4.6 (3.3) | 55 (11.9) | 14.3 (7) | 1.8 (0.5) |
|  | blue | 12.6 (5.7) | 0.4 (0.3) | 0.8 (0.5) | 40.6 (19.9) | 1.6 (0.5) | 40.5 (25.5) | 5.8 (0.9) | 3.9 (1.1) |
| *isotopic and morphological data* | | *δ*^13^C (‰) | *δ*^15^N (‰) | SVL (mm) | HW (mm) | JL (mm) | Mass (g) |  |  |
| vegetation |  |  |  |  |  |  |  |  |  |
|  | grass | -13.3 (0.2) | -1.4 (0.7) | - | - | - | - |  |  |
|  | forb | -26.3 (1.1) | 0.4 (0.4) | - | - | - | - |  |  |
|  | shrub | -27.5 (0.5) | -0.5 (0.6) | - | - | - | - |  |  |
| arthropods |  |  |  |  |  |  |  |  |  |
|  | C_4_ herbivores | -14.7 (0.3) | 2.7 (0.3) | - | - | - | - |  |  |
|  | C_3_ herbivores | -23.5 (1) | 3.7 (0.6) | - | - | - | - |  |  |
|  | non-spider predators | -20.4 (1.1) | 7.9 (0.8) | - | - | - | - |  |  |
|  | spiders | -17.5 (0.5) | 7.9 (0.4) | - | - | - | - |  |  |
| lizards |  |  |  |  |  |  |  |  |  |
| male morph | yellow | -19.5 (0.2) | 7.5 (0.3) | 49.4 (0.8) | 9.3 (0.2) | 9.3 (0.1) | 3 (0.2) |  |  |
|  | orange | -19.1 (0.2) | 5.5 (0.2) | 48.6 (0.8) | 9.2 (0.3) | 8.6 (0.2) | 3.2 (0.2) |  |  |
|  | blue | -19.3 (0.1) | 8.2 (0.4) | 49.6 (0.1) | 8.9 (0.5) | 8.8 (0.2) | 3.7 (0.4) |  |  |

**Figure S1**. Variation in spatial proximity (in metres) of yellow morph *U. ornatus* to either blue (grey bars) or orange (white bars) morph neighbours at a nearby population, clustered by year: 2013 (blue males [n=16], orange males [n=3], yellow males [n=10]) and 2014 (blue males [n=13], orange males [n=3], yellow males [n=20]). Surveys were conducted during June of both years. Yellow morphs were more spatially-clustered around blue males than orange males, regardless of year (although it is clear that these differences were greater in 2013, see also Table S1), supporting our characterisation of this morph as a satellite (see Table S1 for analysis of these data). Bars are mean +1 standard error.


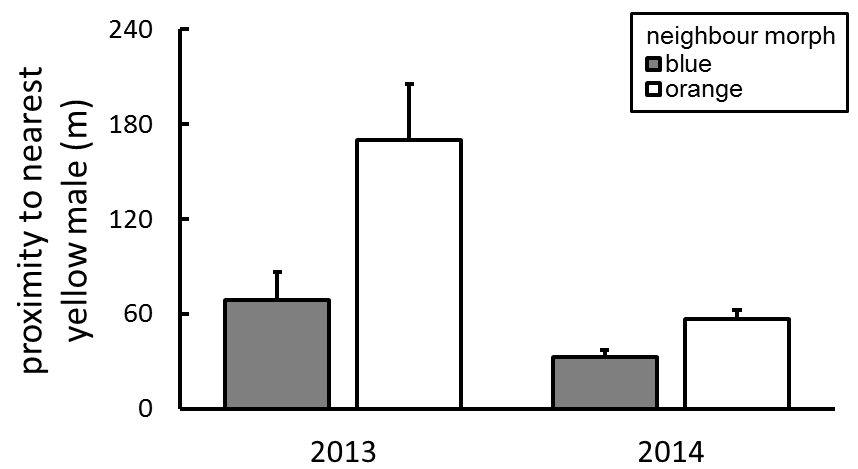


**Figure S2**. Non-metric multidimensional scaling ordination biplot depicting environmental variation between the study sites, measured in 100 m^2^ plots at gridded availability points in each site (n = 32 plots per site). Point shapes differ by site: low-frequency burned site (LB, circles) or high-frequency burned site (HB, squares). Ellipses represent 95% confidence intervals around the centroid for the LB (solid line) and HB (broken line) site. Vectors denote relative direction and magnitude of contribution for each environmental variable that had a significant (*P* < 0.001) fit to the ordination. In general, the LB site had greater forb and bare ground cover, and less grass cover, relative to the HB site.


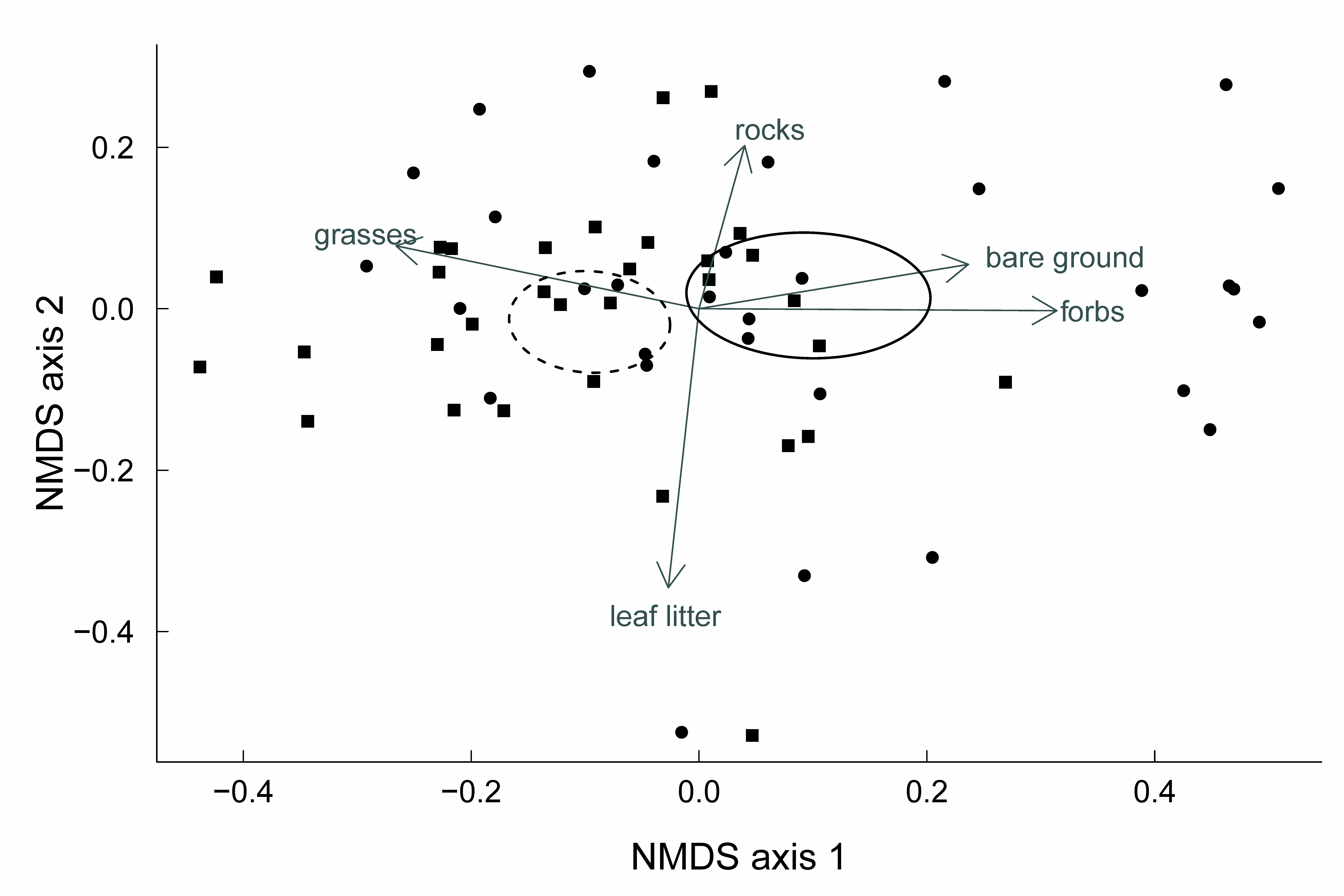


**Figure S3**. Non-metric multidimensional scaling biplot depicting environmental variation between vegetation and structural cover measured at lizard capture (dashed lines, n = 40) and gridded availability (solid lines, n = 32) points in each study site. Point shapes differ by site: low-frequency burned site (LB, circles) or high-frequency burned site (HB, squares). Ellipses represent 95% confidence intervals around the centroid for the low-frequency burned site (LB, grey lines) and high-frequency burned site (HB, black lines). Vectors denote relative direction and magnitude of variation for each environmental variable that had a significant (*P* < 0.001) fit to the ordination. In general, the differences between the habitat characteristics at lizard capture points and those at availability points were enhanced at the HB site compared to the LB site, although lizards at both sites preferred microhabitats with higher leaf litter and forb cover.


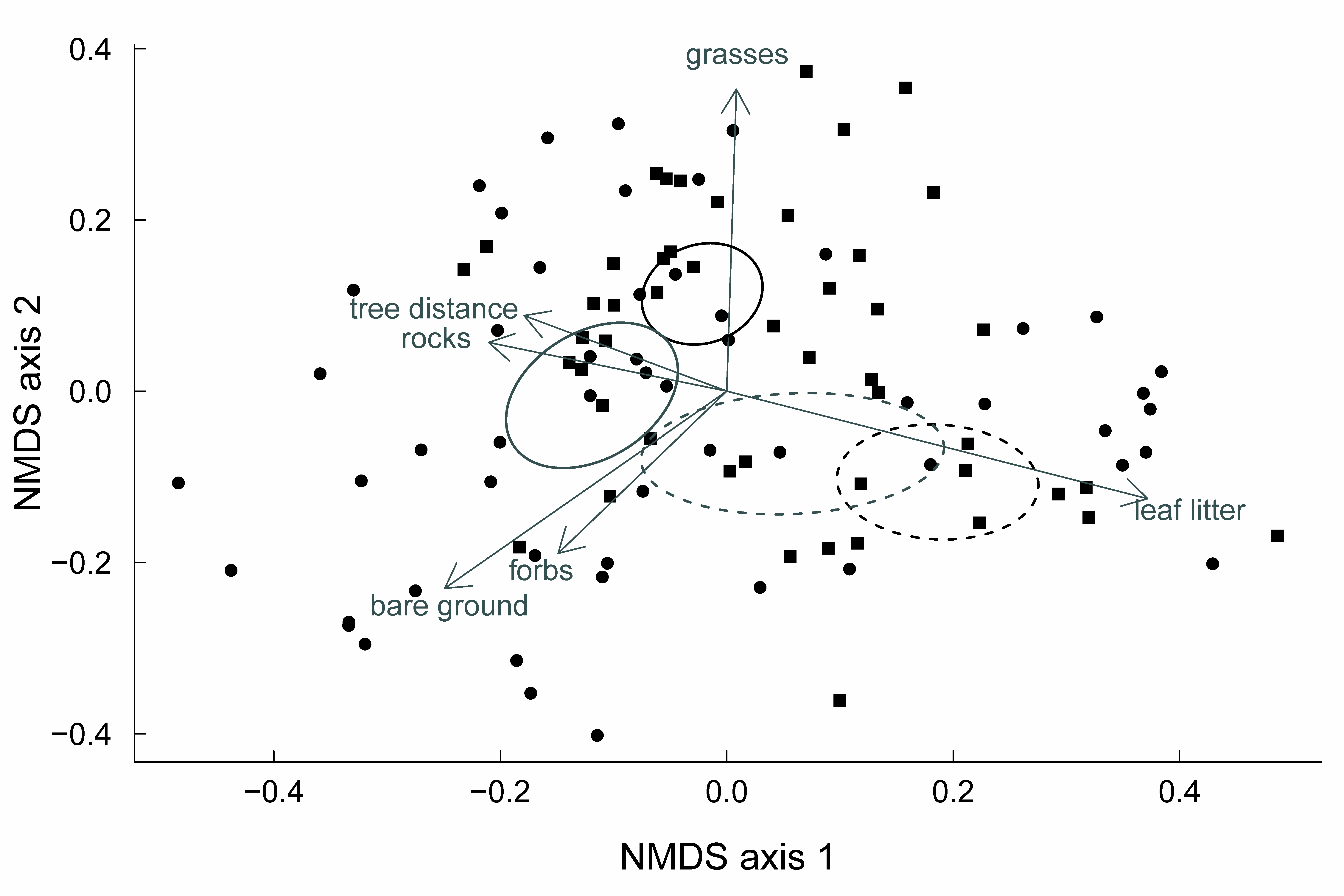

Supplement: Table S1. Results of analysis of yellow male U. ornatus satellite behaviour. Table S2 - Summary of vegetation, arthropod, and U. ornatus datasets used in this study. Figure S1 - Variation in spatial proximity (in metres) of yellow morph U. ornatus to either blue or orange males from a nearby populat [file rsos150531supp1.docx]
